# Supplementary material for: The O-glycosyltransferase C1GALT1 promotes EWSR1::FLI1 expression and is a therapeutic target for Ewing sarcoma
Source: Nat Commun. 2025 Feb 2;16:1267. doi: 10.1038/s41467-025-56632-0 (PMC11788431; doi:10.1038/s41467-025-56632-0)
Supplement: Supplementary file 1 — Supplementary Information [file 41467_2025_56632_MOESM1_ESM.pdf]

Supplementary Information

The O-glycosyltransferase C1GALT1 promotes *EWSR1::FLI1* expression and is a therapeutic target for Ewing sarcoma

Shahid Banday[1, *], Alok K. Mishra[1], Romana Rashid[2], Tianyi Ye[1], Amjad Ali[1], Junhui Li[1], Jason T. Yustein[4], Michelle A. Kelliher[1], Lihua Julie Zhu[1,3], Sara K. Deibler[1], Sunil K. Malonia[1,*] & Michael R. Green[1]

Supplementary Figure 1

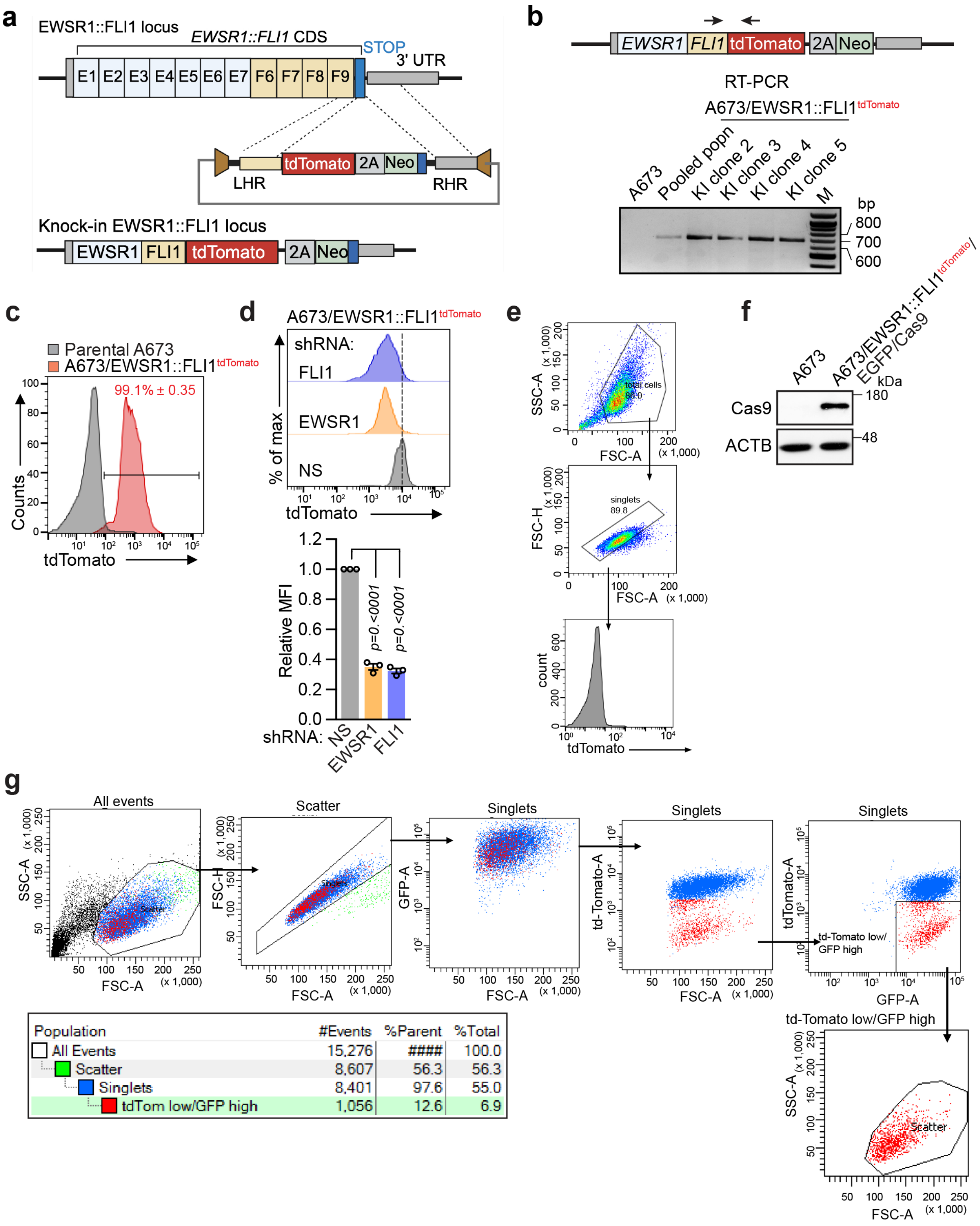

Supplementary Fig. 1 | Construction and validation of the A673/EWSR1::FLI1^{tdTomato}/ EGFP/Cas9 reporter cell line. a Schematic detailing the CRISPR/Cas9-mediated homology-directed repair strategy to insert the tdTomato reporter at the 3' end of the endogenous

EWSR1::FLI1 gene. LHR, left homology region; RHR, right homology region; 2A, 2A self-cleaving peptide; Neo, neomycin resistance cassette. **b** (Top) Schematic of the RT-PCR strategy to confirm insertion of tdTomato at the 3' end of *FLI1*. Arrows indicate the location of the primer pairs. (Bottom) RT-PCR analysis detecting the FLI1-tdTomato fusion in the A673/EWSR1::FLI1^{tdTomato} pooled population and three single A673/EWSR1::FLI1^{tdTomato} knock-in (KI) clones. Parental A673 cells are shown as a negative control. The experiment was performed once. The KI clone 2 was selected for the reporter cell line. **c** Representative flow cytometry histogram (from $n = 2$ biologically independent experiments) showing the percentage of tdTomato-positive cells in the A673/EWSR1::FLI1^{tdTomato} population (red). Parental A673 cells (gray) are shown as a negative control. The results show that the vast majority of the A673/EWS-FLI1^{tdTomato} cell population is tdTomato-positive. **d** Flow cytometry analysis of tdTomato in A673/EWSR1::FLI1^{tdTomato} cells stably expressing a non-silencing (NS), EWSR1 or FLI1 shRNA. Top, representative histogram. Bottom, quantification of mean fluorescence intensity (MFI). The results were normalized to the MFI observed in cells expressing the control NS shRNA, which was set to 1. Data are presented as mean ± SEM ($n = 3$ biologically independent experiments). *P* values were calculated using one-way ANOVA with post-hoc Dunnett's multiple comparisons test. **e** Flow cytometry gating strategy used for (c) and (d). FSC, forward scatter; SSC, side scatter. **f** Representative immunoblot (from $n = 2$ biologically independent experiments) confirming Cas9 expression in A673/EWSR1::FLI1^{tdTomato}/EGFP/Cas9 cells. Parental A673 cells are shown as a negative control. β-actin (ACTB) was monitored as a loading control. **g** FACS gating/sorting strategy used for the CRISPR screen. From the total live singlets, cells that were positive for GFP were pre-gated. Within the GFP-positive subset, the cells expressing low levels of tdTomato were gated and subsequently sorted. Source data are provided as a Source Data file.

Supplementary Figure 2

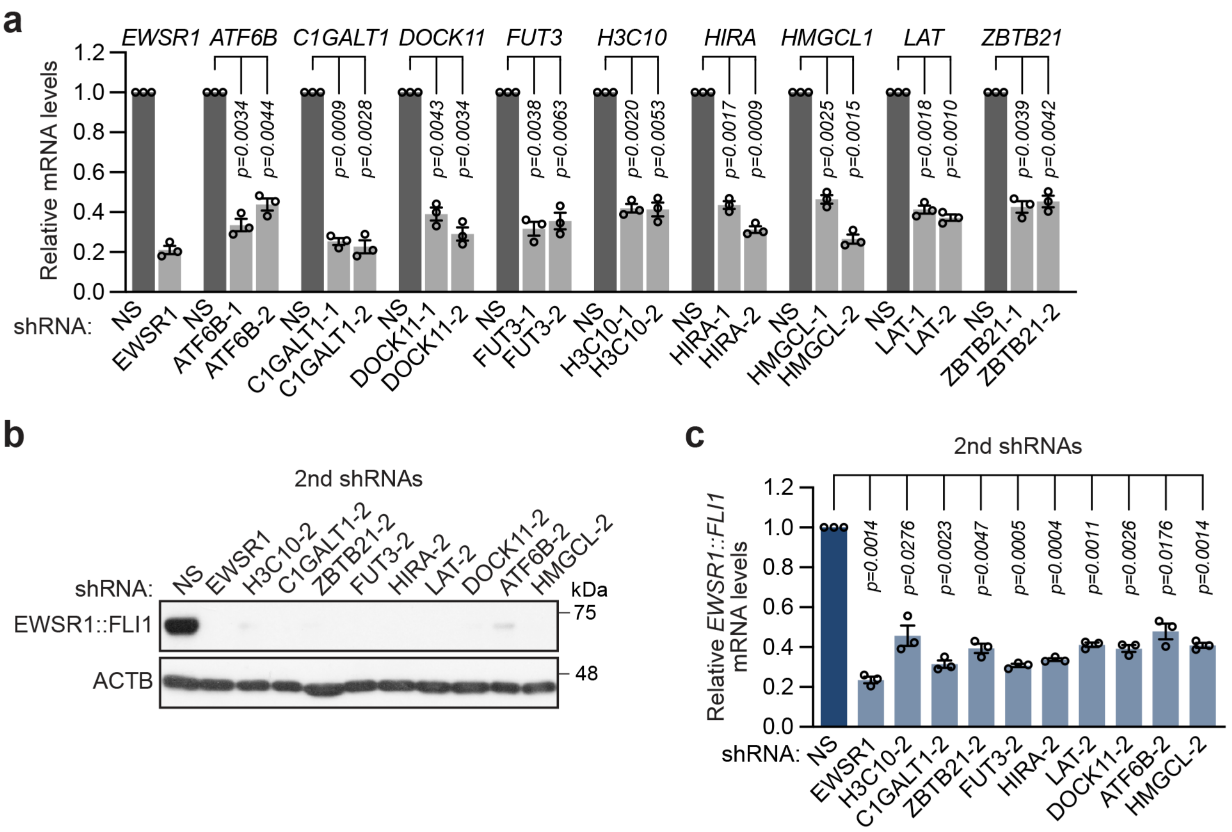

Supplementary Fig. 2 | Validation of candidates from the primary screen using a second, independent shRNA. a qRT-PCR analysis monitoring knockdown efficiency of shRNAs targeting each of the nine candidates in A673 cells. The results were normalized to that obtained with a NS shRNA, which was set to 1. Data are presented as mean ± SEM (n = 3 biologically independent experiments). P values were calculated using one-way ANOVA with post-hoc Dunnett's multiple comparisons test. **b** Representative immunoblot (from n = 3 biologically independent experiments) showing EWSR1::FLI1 protein levels (monitored using an anti-FLI1 antibody) in A673 cells stably expressing a second shRNA, unrelated to that used in Fig. 1c, targeting each of the nine validated candidates. **c** qRT-PCR analysis monitoring relative *EWSR1::FLI1* mRNA levels in A673 cells stably expressing a second shRNA, unrelated to that used in Fig. 1d, targeting each of the nine validated candidates. Data are presented as mean ± SEM (n = 3 biologically independent experiments). P values were calculated using one-way ANOVA with post-hoc Dunnett's multiple comparisons test. Source data are provided as a Source Data file.

Supplementary Figure 3

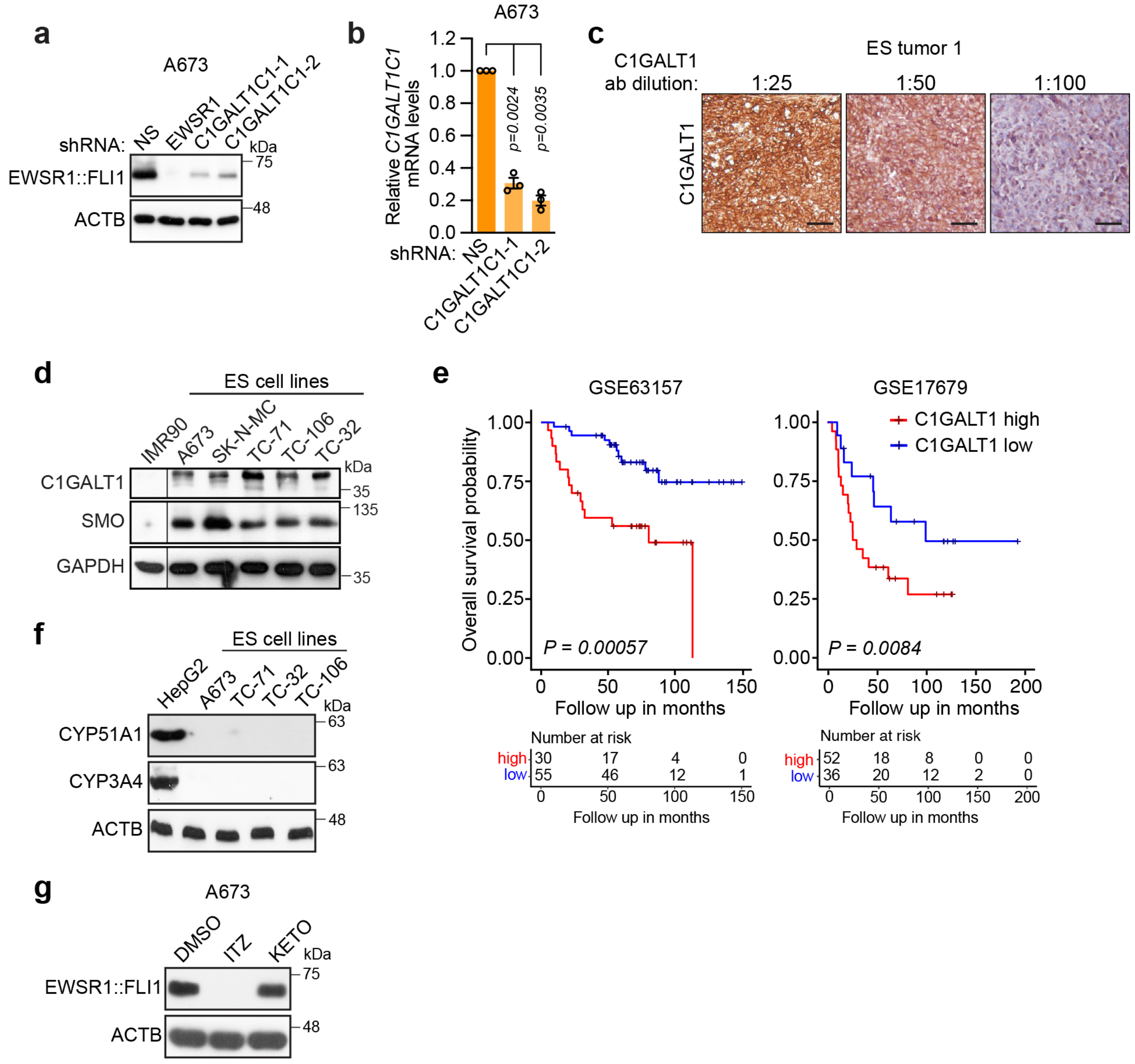

Supplementary Fig. 3 | Additional experiments and analyses related to Figure 2. a Representative immunoblot (from n = 3 biologically independent experiments) showing EWSR1::FLI1 levels (monitored using an anti-FLI1 antibody) in A673 cells stably expressing a NS, EWSR1 or one of two independent C1GALT1C1 shRNAs. **b** qRT-PCR analysis monitoring knockdown efficiency of the C1GALT1C1 shRNAs. Data are presented as mean ± SEM (n = 3 biologically independent experiments). P values were calculated using one-way ANOVA with post-hoc Dunnett's multiple comparisons test. **c** Representative IHC images showing C1GALT1 staining in an ES patient tumor sample following incubation with different dilutions of the anti-C1GALT1 antibody (ab). The results show that a specific C1GALT1 signal was achieved using a dilution of 1:100, which was used in the IHC experiments of Figure 2c. **d** Immunoblot monitoring C1GALT1 and SMO levels in human ES cell lines or, as a negative control, IMR90 fibroblasts. The experiment was performed once. The vertical line indicates lanes removed from the contiguous blot. GAPDH was monitored as a loading control and was run on a separate gel than

that used to blot for C1GALT1 due to the similarity in the molecular weight of C1GALT1 (42 kDa) and GAPDH (37 kDa). **e** Kaplan-Meier analysis showing reduced overall survival probability in ES patients with high C1GALT1 expression compared to those with low C1GALT1 expression, derived from the datasets GSE17679 and GSE63157. *P* values were calculated using log-rank test. **f** Immunoblots showing CYP51A1 and CYP3A4 levels in human ES cell lines or, as a positive control, HepG2 cells. The experiment was performed once. The results show that CYP51A1 and CYP3A4 were not detectable in ES cell lines. **g** Immunoblot showing EWSR1::FLI1 levels (monitored using an anti-FLI1 antibody) in A673 cells treated with 100 nM ITZ or 10 μM ketoconazole (KETO) for 72 hours. The experiment was performed once. The results show that ketoconazole, an FDA-approved anti-fungal CYP51A1 inhibitor, does not reduce EWSR1::FLI1 levels. Collectively, the results of e and f rule out the possibility that ITZ-mediated reduction in EWSR1::FLI1 occurs through CYP51A1/CYP3A4 inhibition. Source data are provided as a Source Data file.

Supplementary Figure 4

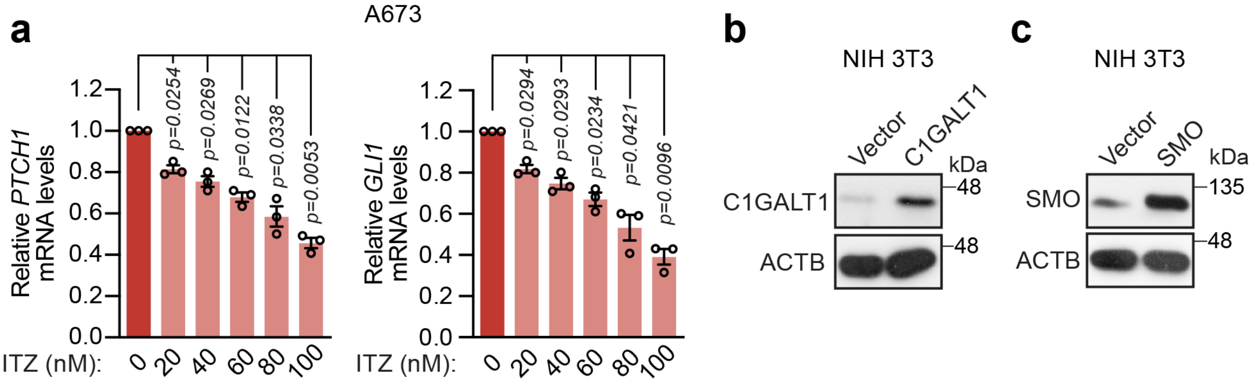

Supplementary Fig. 4 | Additional experiments related to Figure 4. a qRT-PCR analysis monitoring relative *PTCH1* and *GLI1* mRNA levels in A673 cells treated with DMSO or ITZ (100 nM for 72 hours). Data are presented as mean ± SEM (n = 3 biologically independent experiments). *P* values were calculated using one-way ANOVA with post-hoc Dunnett's multiple comparisons test. **b** Representative immunoblot (from n = 2 biologically independent experiments) confirming elevated C1GALT1 levels in NIH 3T3 cells transduced with a C1GALT1-expression plasmid. Empty vector is shown as a control. **c** Representative immunoblot (from n = 2 biologically independent experiments) confirming elevated SMO levels in NIH 3T3 cells transduced with a SMO-expression plasmid. Empty vector is shown as a control. Source data are provided as a Source Data file.

Supplementary Figure 5

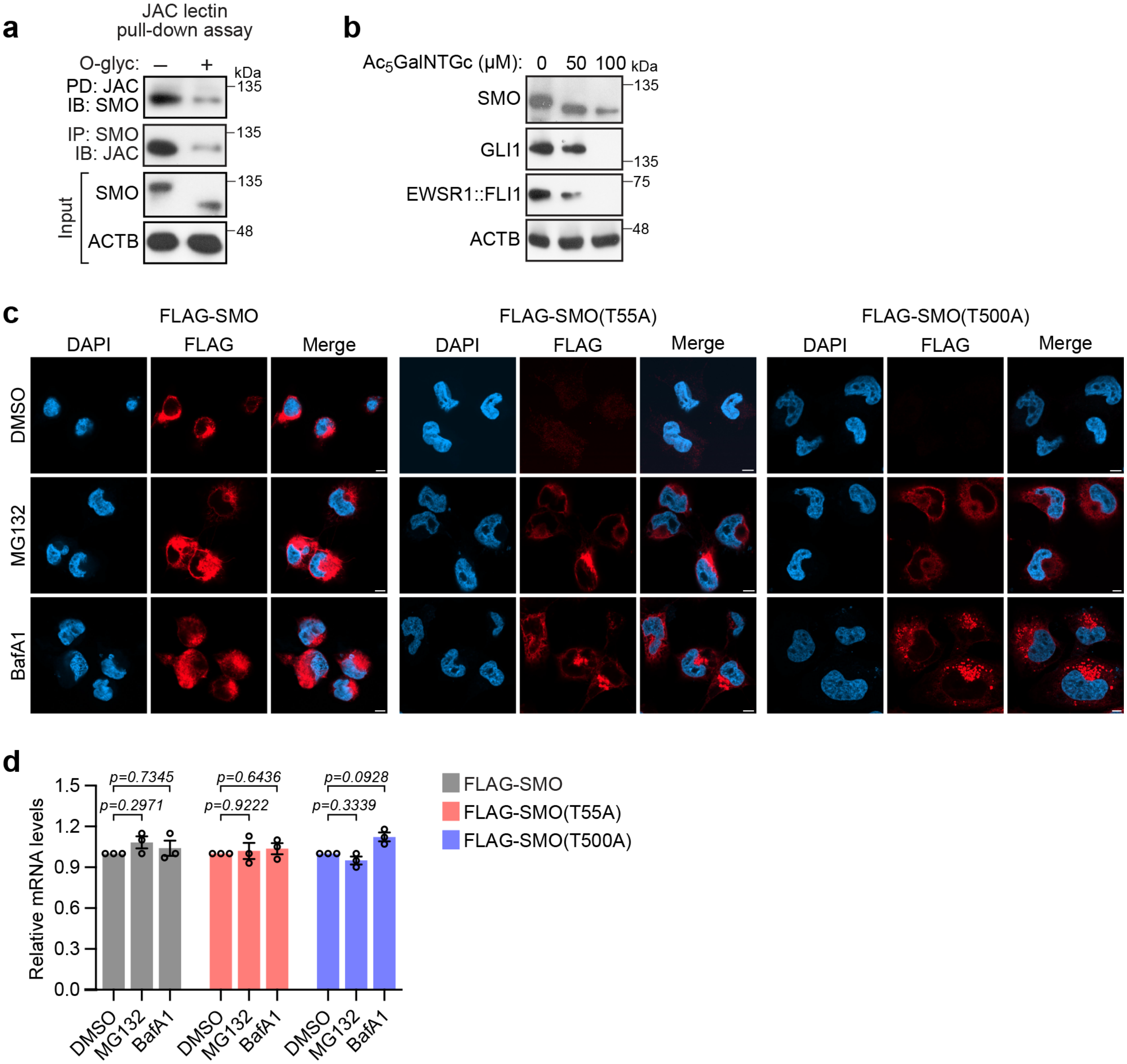

Supplementary Fig. 5 | Additional experiments related to Figure 5. a Jacalin (JAC) lectin pull-down assay. Representative immunoblot (from n = 2 biologically independent experiments) showing the level of O-glycosylated SMO, detected by either immunoblot (IB) analysis for SMO in a JAC lectin pull-down (PD) assay or IB analysis for JAC in a SMO immunoprecipitate (IP), in A673 cell lysates following treatment with a combination of O-glycosidase and α2-3,6,8 neuraminidase, which removes terminal sialic residues that can inhibit binding to lectins and cleavage by O-glycosidase. The level of total SMO in whole cell lysate (input) is also shown. **b** Representative immunoblot (from n = 2 biologically independent experiments) monitoring SMO, GLI1 and EWSR1::FLI1 levels in A673 cells treated with either DMSO or Ac_5GalNTGc. **c** Representative immunofluorescence microscopy images (from n = 2 biologically independent experiments) showing the levels of FLAG-SMO, FLAG-SMO(T55A) or FLAG-SMO(T500A) (red) in A673 cells treated with DMSO, MG132 or bafilomycin A1 (BafA1). DAPI staining is

shown in blue. Scale bars, 5 μm. The results independently confirm that the SMO(T55A) and SMO(T500A) mutant proteins were expressed at low or undetectable levels, and that their expression could be restored by addition of MG132 or BafA1. **d** qRT-PCR analysis monitoring relative steady state mRNA levels of FLAG-SMO, SMO(T55A) and SMO(T500A) in A673 cells treated with DMSO, MG132 or BafA1. Expression of FLAG-SMO was detected using a primer pair spanning the junction between FLAG and SMO. The results were normalized to that obtained with FLAG-SMO in DMSO-treated cells, which was set to 1. Data are presented as mean ± SEM (n = 3 biologically independent experiments). P values were calculated using one-way ANOVA with post-hoc Dunnett's multiple comparisons test. The results show that the steady state mRNA levels of FLAG-SMO, SMO(T55A) and SMO(T500A) were unaffected by treatment with MG132 or BafA1. Source data are provided as a Source Data file.

Supplementary Figure 6

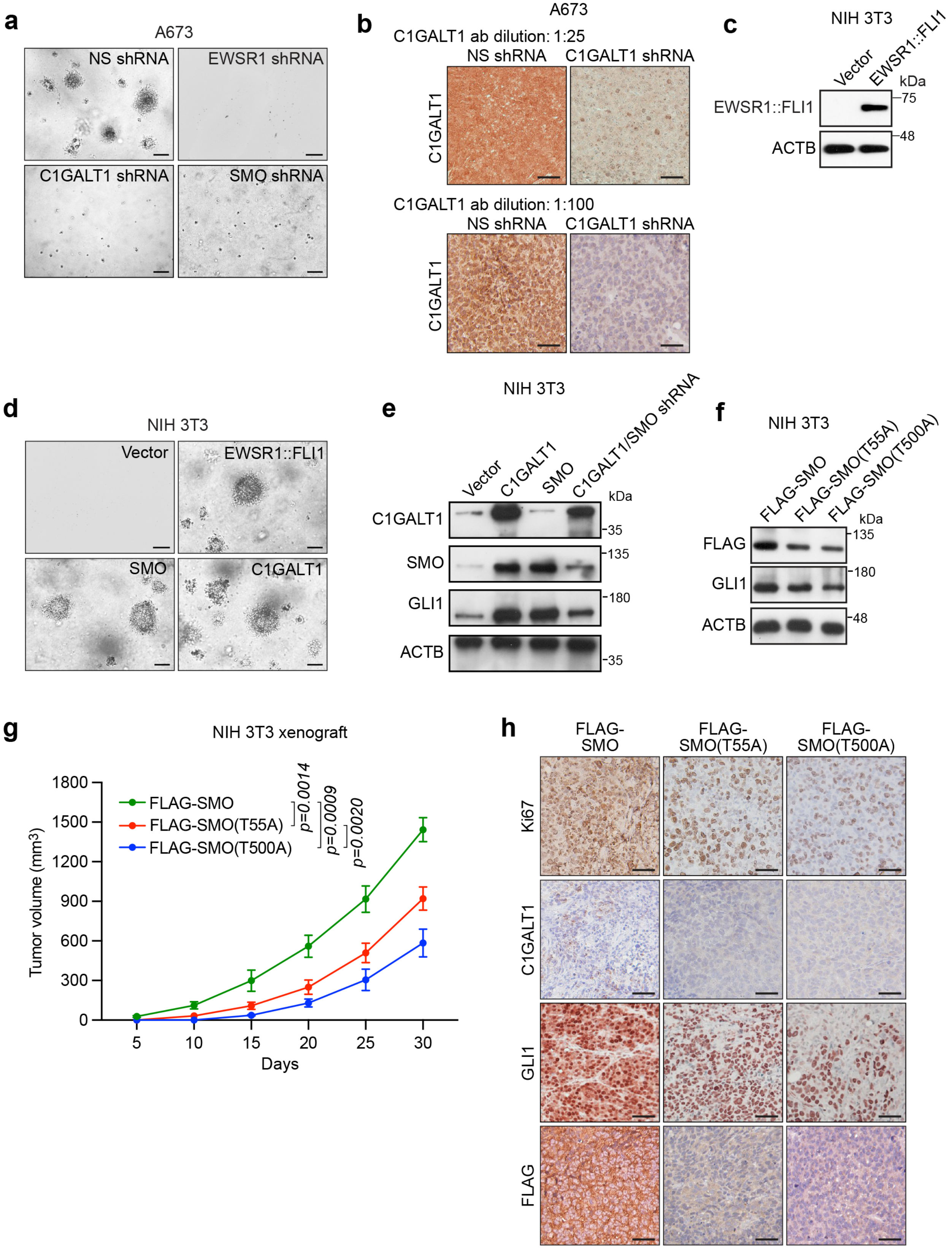

Supplementary Fig. 6 | Control experiments related to Figure 6. a Soft agar assay. Representative images (from *n* = 3 biologically independent experiments) showing colony formation of A673 cells stably expressing a NS, EWSR1, C1GALT1 or SMO shRNA. Scale bars, 20 µm. **b** Representative IHC images showing C1GALT1 staining in mouse A673 tumor xenografts following incubation with different dilutions of the anti-C1GALT1 antibody (ab). The results show that a specific C1GALT1 signal was achieved using a dilution of 1:100, which was used in all mouse tumor IHC experiments. **c** Representative immunoblot (from *n* = 2 biologically independent experiments) confirming elevated EWSR1::FLI1 levels (monitored using an anti-FLI1 antibody) in NIH 3T3 cells transduced with an EWSR1::FLI1-expression plasmid. Empty vector is shown as a negative control. **d** Soft agar assay. Representative images (from *n* = 3 biologically independent experiments) showing colony formation of NIH 3T3 cells expressing empty vector, EWSR1::FLI1, SMO or C1GALT1. Scale bars, 20 µm. **e** Representative immunoblot (from *n* = 2 biologically independent experiments) showing levels of C1GALT1, SMO and GLI1 in NIH 3T3 cells ectopically expressing vector, C1GALT1 or SMO, or cells ectopically expressing C1GALT1 and a SMO shRNA. **f** Representative immunoblot (from *n* = 3 biologically independent experiments) showing levels of FLAG-SMO in NIH 3T3 cells ectopically expressing FLAG-SMO, FLAG-SMO(T55A) or FLAG-SMO(T500A). Also shown are the levels of GLI1. The results show that the levels of FLAG-SMO(T55A) and FLAG-SMO(T500A) are reduced relative to FLAG-SMO in NIH 3T3 cells, with an accompanying decrease in GLI1 levels, indicating reduced Hh signaling. **g** Tumor formation of NIH 3T3 cells ectopically expressing FLAG-SMO, FLAG-SMO(T55A) or FLAG-SMO(T500A) following subcutaneous injection into NSG mice. Tumor dimensions were monitored every 5 days starting at day 5, when tumors became palpable. Data are presented as mean ± SD (*n* = 5 mice per group). *P* values were calculated at day 30 using two-way ANOVA with post-hoc Tukey's multiple comparisons test. The results show that NIH 3T3 cells expressing FLAG-SMO(T55A) or FLAG-SMO(T500A) had reduced tumor growth relative to those expressing wild-type FLAG-SMO. **h** Representative IHC images showing Ki67, C1GALT1, GLI1 and FLAG and staining in tumors derived from mice in (g) at day 30. Scale bar, 50 µm. The results confirmed reduced GLI1 and FLAG staining in tumors derived from FLAG-SMO(T55A) or FLAG-SMO(T500A)-expressing cells. Source data are provided as a Source Data file.

Supplementary Figure 7

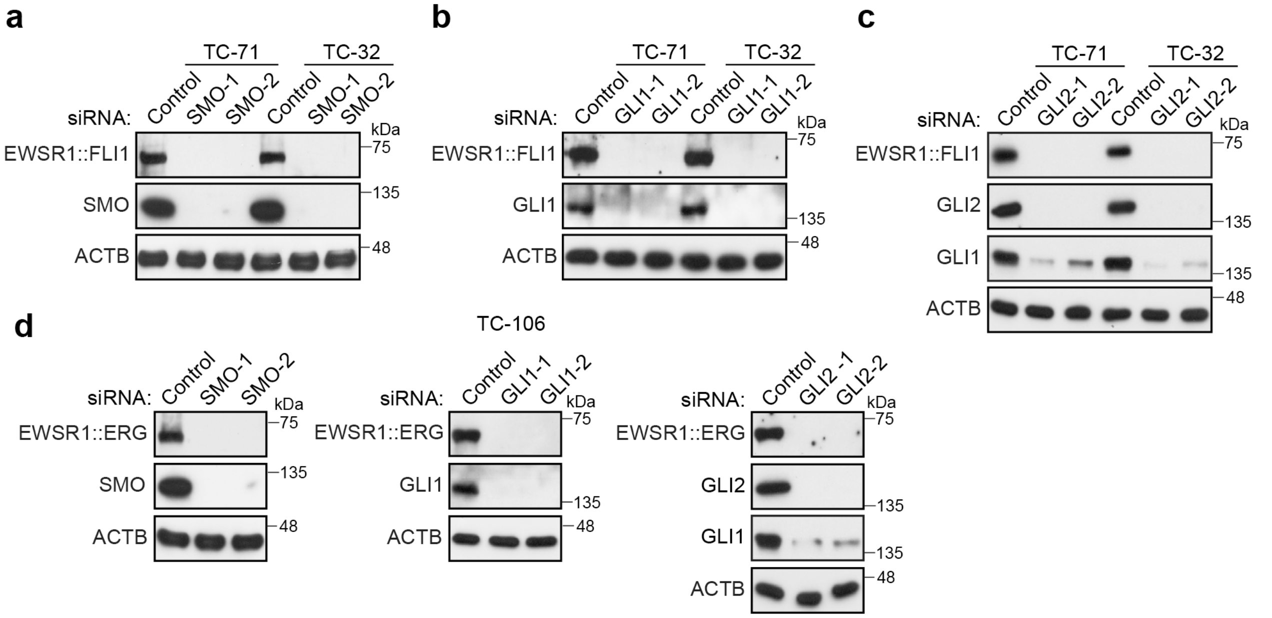

Supplementary Fig. 7 | Confirmation that Hh signaling is required for expression of EWSR1::FLI1/ERG in additional human ES cell lines. a-c Representative immunoblot (from *n* = 3 biologically independent experiments) showing EWSR1::FLI1 protein levels (monitored using an anti-FLI1 antibody) in TC-71 and TC-32 cells expressing a control siRNA or one of two independent SMO (**a**), GLI1 (**b**) or GLI2 (**c**) siRNAs. **d** Representative immunoblot (from *n* = 3 biologically independent experiments) showing EWSR1::ERG protein levels (monitored using an anti-EWSR1 antibody) in TC-106 cells expressing a control or SMO (left), GLI1 (middle) or GLI2 (right) siRNA.

Supplementary Figure 8

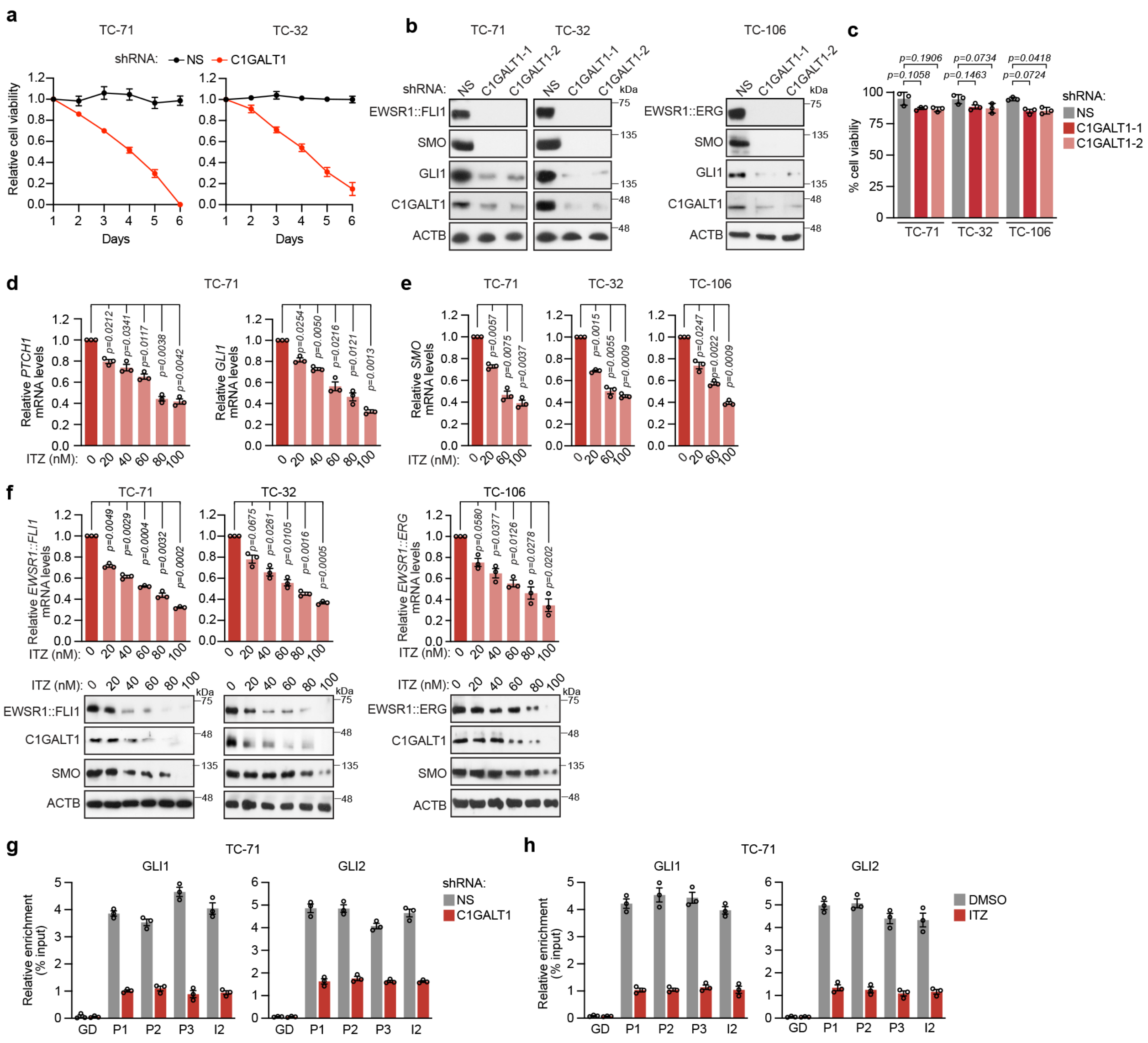

Supplementary Fig. 8 | Confirmation that C1GALT1 promotes Hh signaling and EWSR1::FLI1/ERG expression in additional human ES cell lines. a Cell viability, monitored by PrestoBlue assay, of TC-71 and TC-32 cells following transduction with a lentivirus expressing a NS or C1GALT1 shRNA and drug selection for 2 days. Data are presented as mean ± SD (n = 3 biologically independent experiments). **b** (Left) Representative immunoblot (from n = 3 biologically independent experiments) showing EWSR1::FLI1 protein levels in TC-71 and TC-32 cells (monitored using an anti-FLI1 antibody) or EWSR1::ERG protein levels in TC-106 cells (monitored using an anti-EWSR1 antibody), as well as SMO, GLI1 and C1GALT1, following transduction with a high titer lentivirus expressing a NS or C1GALT1 shRNA. **c** Cell viability, monitored by trypan blue exclusion assay, of TC-71, TC-32 and TC-106 cells following transduction with a high titer lentivirus expressing a NS or C1GALT1 shRNA. Data are presented as mean ± SD (n = 3 biologically independent experiments). P values were calculated using one-way ANOVA with post-hoc Dunnett's multiple comparisons test. **d** qRT-PCR analysis monitoring relative *PTCH1* (left) or *GLI1* (right) mRNA levels in TC-71 cells following treatment with DMSO or ITZ for 72 hours. Data are presented as mean ± SEM (n = 3 biologically independent

experiments). *P* values were calculated using one-way ANOVA with post-hoc Dunnett's multiple comparisons test. **e** qRT-PCR analysis monitoring relative *SMO* mRNA levels in TC-71, TC-32 and TC-106 cells following treatment with DMSO or ITZ for 48 hours. Data are presented as mean ± SEM (n = 3 biologically independent experiments). *P* values were calculated using one-way ANOVA with post-hoc Dunnett's multiple comparisons test. **f** (Top) qRT-PCR analysis monitoring relative *EWSR1::FLI1* mRNA levels in TC-71 and TC-32 cells, and *EWSR1::ERG* mRNA levels in TC-106 cells, following treatment with DMSO or ITZ for 48 hours. Data are presented as mean ± SEM (n = 3 biologically independent experiments). *P* values were calculated using one-way ANOVA with post-hoc Dunnett's multiple comparisons test. (Bottom) Representative immunoblot (from n = 3 biologically independent experiments) showing EWSR1::FLI1 protein levels (monitored using an anti-FLI1 antibody) in TC-71 and TC-32 cells, or EWSR1::ERG protein levels (monitored using an anti-EWSR1 antibody) in TC-106 cells as well as C1GALT1 and SMO following treatment with DMSO or ITZ for 48 hours. **g, h** ChIP analysis monitoring binding of GLI1 and GLI2 to *EWSR1* regions containing GLI-binding motifs or, as a negative control, a gene desert (GD) region, in TC-71 cells following transduction with a high titer lentivirus expressing a NS or C1GALT1 shRNA (**g**) or following treatment with DMSO or ITZ (100 nM for 48 hours) (**h**). Data are presented as mean ± SEM (n = 3 biologically independent experiments). Source data are provided as a Source Data file.

Supplementary Figure 9

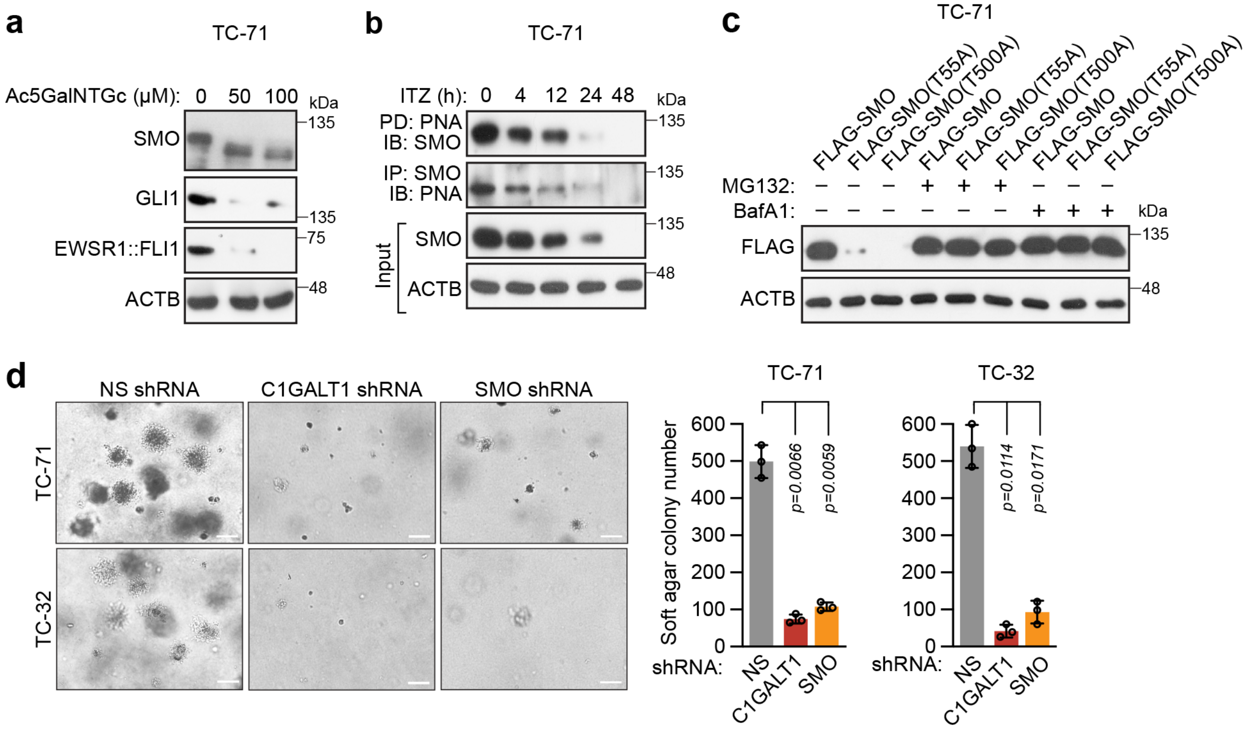

Supplementary Fig. 9 | Confirmation the SMO is O-glycosylated, and that C1GALT1 is required for EWSR1::FLI1/ERG-mediated transformation, in additional ES cell lines. a Representative immunoblot (from *n* = 2 biologically independent experiments) monitoring SMO, GLI1 and EWSR1::FLI1 levels in TC-71 cells treated with either DMSO or Ac_5GalNTGc. **b** PNA lectin pull-down assay. Representative immunoblot (from *n* = 2 biologically independent experiments) showing the level of O-glycosylated SMO, detected by either immunoblot (IB) analysis for SMO in a PNA lectin pull-down (PD) assay or IB analysis for PNA in a SMO immunoprecipitate (IP), in TC-71cell lysates following treatment with DMSO or 100 nM ITZ for the indicated times. The level of total SMO in whole cell lysate (input) is also shown. **c** Representative immunoblot (from *n* = 2 biologically independent experiments) showing FLAG-SMO levels in TC-71 cells stably expressing FLAG-tagged derivatives of wild-type SMO or SMO(T55A) or SMO(T500A) mutant in the presence or absence of MG132 or BafA1. **d** Soft agar assay monitoring colony formation of TC-71 and TC-32 cells following transduction with a high titer lentivirus expressing a NS or C1GALT1 shRNA. Data are presented as mean ± SD (*n* = 3 biologically independent experiments). *P* values were calculated using one-way ANOVA with post-hoc Dunnett's multiple comparisons test. Source data are provided as a Source Data file.

Supplementary Figure 10

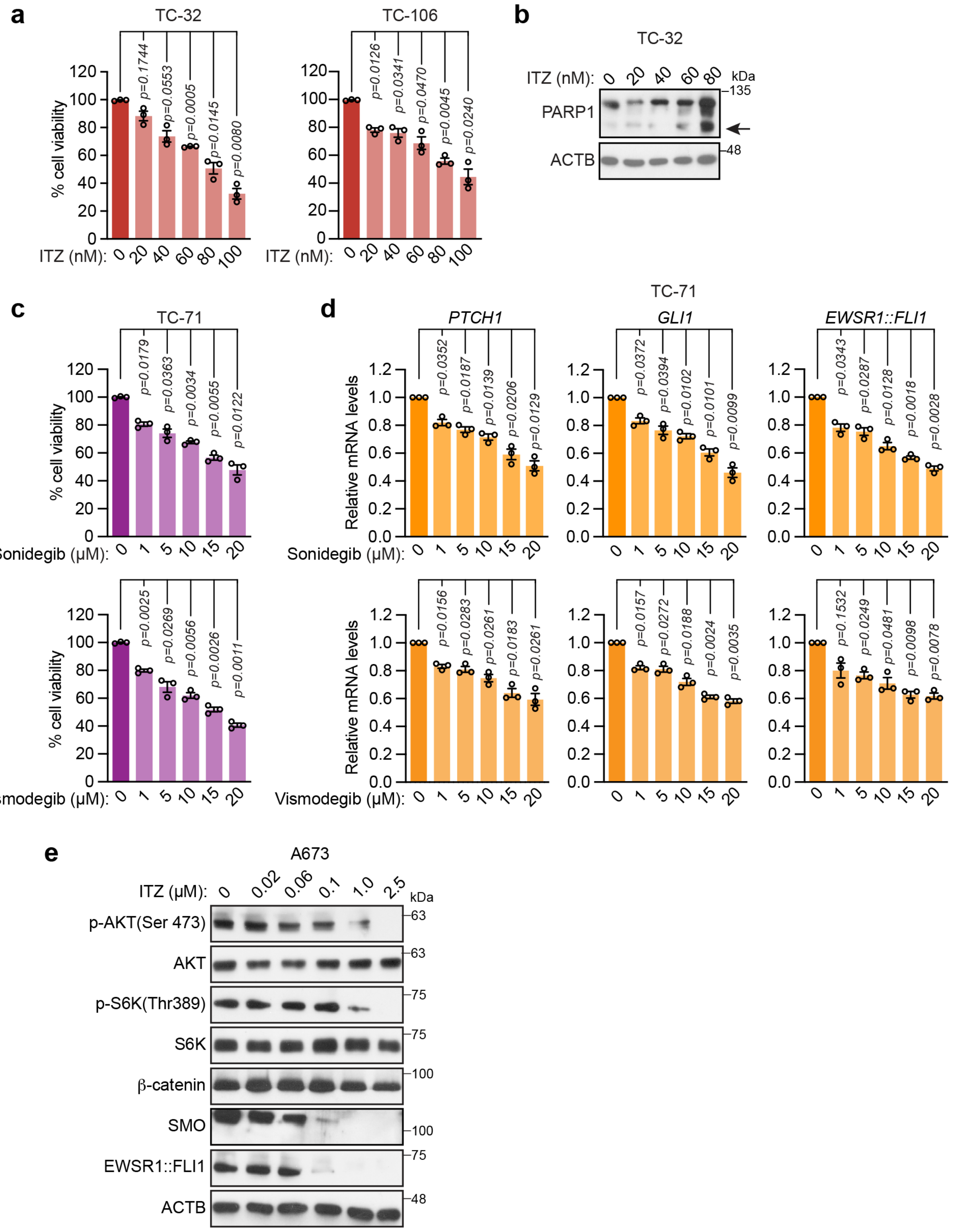

Supplementary Fig. 10 | Additional experiments related to Figure 7. a Cell viability, as monitored by PrestoBlue assay, of TC-32 and TC-106 cells treated with DMSO or ITZ for 72 hours. Data are presented as mean ± SEM (*n* = 3 biologically independent experiments). *P* values

were calculated using one-way ANOVA with post hoc Dunnett's multiple comparisons test. **b** Representative immunoblot (from *n* = 3 biologically independent experiments) showing PARP levels in TC-32 cells treated with DMSO or ITZ for 48 hours. Cleaved PARP is indicated by the arrow. **c** Cell viability, as monitored by PrestoBlue assay, of TC-71 cells treated with sonidegib (top) or vismodegib (bottom) for 72 hours. Data are presented as mean ± SEM (*n* = 3 biologically independent experiments). *P* values were calculated using one-way ANOVA with post-hoc Dunnett's multiple comparisons test. **d** qRT-PCR analysis monitoring relative *PTCH1*, *GLI1* and *EWSR1::FLI1* mRNA levels in TC-71 cells treated with DMSO or sonidegib (top) or vismodegib (bottom) for 72 hours. Data are presented as mean ± SEM (*n* = 3 biologically independent experiments). *P* values were calculated using one-way ANOVA with post-hoc Dunnett's multiple comparisons test. **e** Representative immunoblots (from *n* = 3 biologically independent experiments) showing levels of phosphorylated AKT (p-AKT(Ser473)), total AKT, phosphorylated S6K (p-S6K(Thr389)), β-catenin, SMO and EWSR1::FLI1 (monitored using an anti-FLI1 antibody) in A673 cells treated with DMSO or ITZ for 72 hours. Source data are provided as a Source Data file.

Supplementary Figure 11

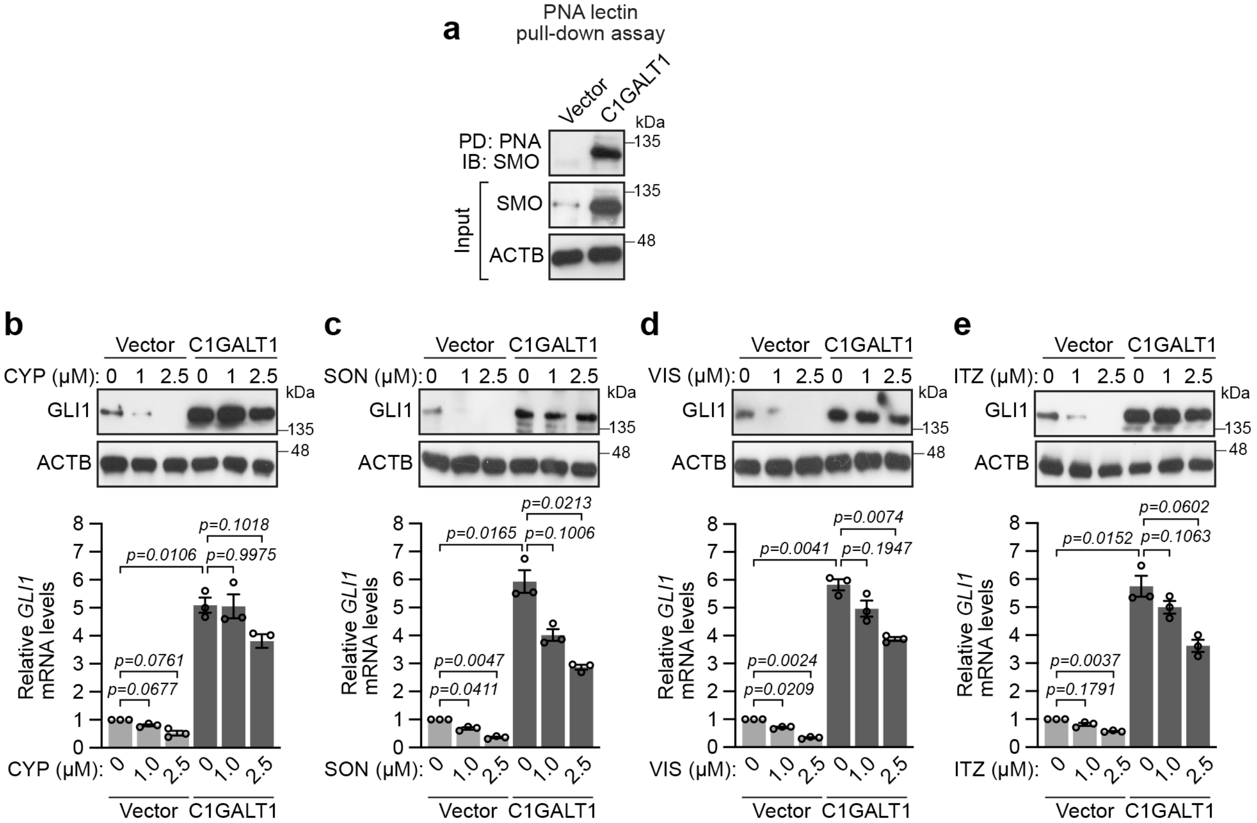

Supplementary Fig. 11 | Ectopic expression of C1GALT1 in NIH3T3 cells abrogates the decrease in GLI1 observed upon treatment with conventional SMO inhibitors. a Representative immunoblot (from *n* = 3 biologically independent experiments) showing levels of O-glycosylated SMO (monitored by a PNA lectin pull-down assay) and total SMO in NIH 3T3 cells expressing vector or C1GALT1. **b-e** (Top) Representative immunoblot (from *n* = 3 biologically independent experiments) monitoring GLI1 levels in NIH 3T3 cells expressing vector or C1GALT1 and treated with cyclopamine (CYP), sonidegib (SON), vismodegib (VIS) and itraconazole (ITZ) for 48 hours. (Bottom) qRT-PCR analysis monitoring relative *GLI1* mRNA levels. Data are presented as mean ± SEM (*n* = 3 biologically independent experiments). *P* values were calculated using one-way ANOVA with post-hoc Dunnett's multiple comparisons test. Source data are provided as a Source Data file.

Supplementary Figure 12

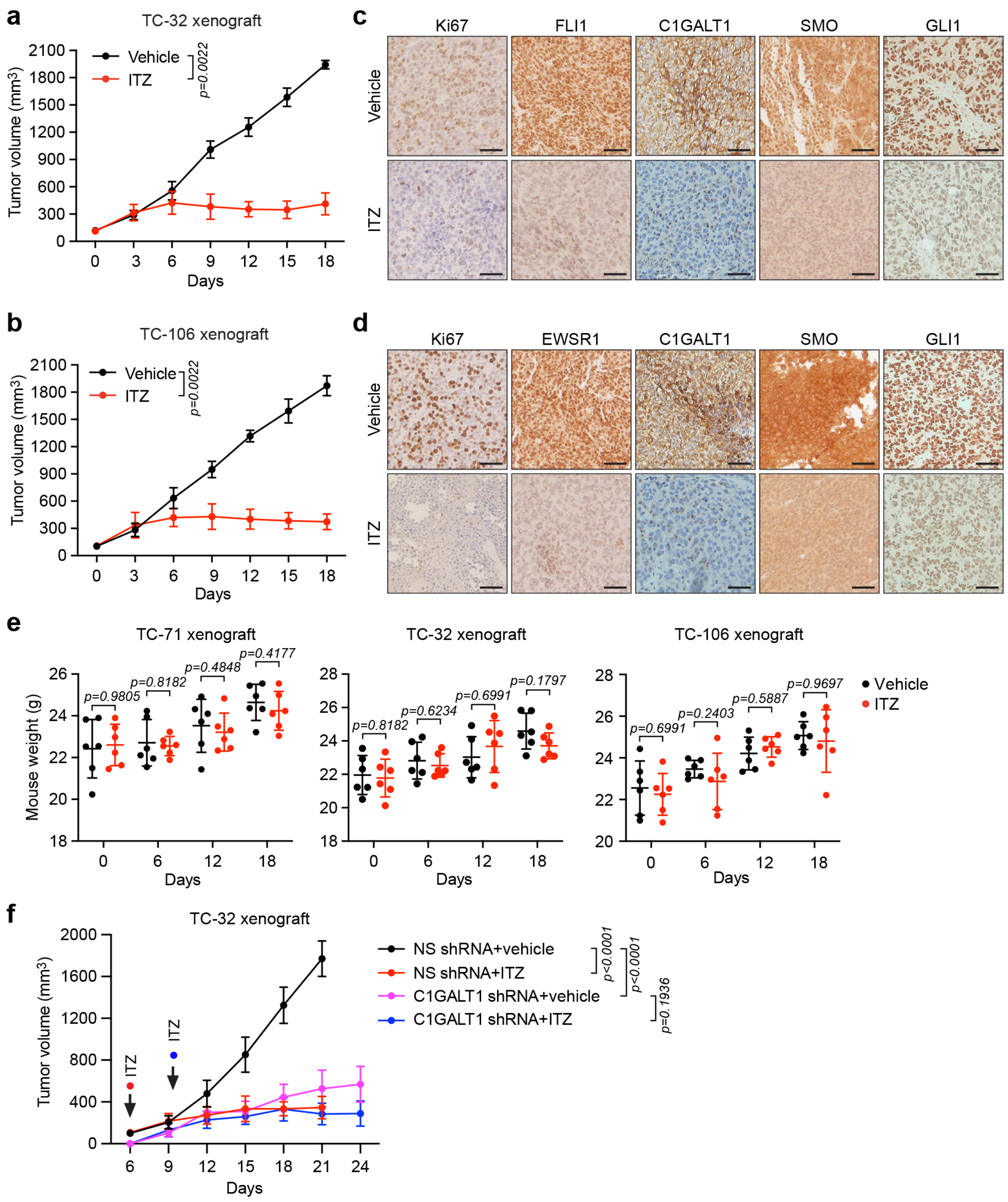

Supplementary Fig. 12 | Confirmation of ITZ-mediated reduction in tumor formation using additional ES cell lines. a, **b** Tumor formation. TC-32 (**a**) or TC-106 (**b**) cells were injected subcutaneously into NSG mice and when tumors reached ~100 mm^3 (denoted as day 0), mice were treated daily with vehicle or ITZ (100 mg/kg by oral gavage) and tumor dimensions were

monitored every 3 days. Data are presented as mean ± SD (*n* = 6 mice per group). *P* values were calculated at day 18 using two-tailed Mann-Whitney test. **c, d** Representative IHC images showing Ki67, FLI1, C1GALT1, SMO and GLI1 staining in TC-32 (**c**) or TC-106 (**d**) xenografts from vehicle- or ITZ-treated mice following 18 days of treatment. Scale bar, 20 µm. **e** Animal weight of mice injected with ES cell lines and treated with vehicle or ITZ. Data are presented as mean ± SD (*n* = 6 mice per group). *P* values were calculated using two-tailed Mann-Whitney test. The results show that the dose of ITZ used in these experiments is well tolerated. **f** Tumor formation. TC-32 cells, following transduction with a high titer lentivirus expressing a NS or C1GALT1 shRNA, were injected subcutaneously into NSG mice and when tumors reached ~100 mm^3 (day 6 for NS shRNA-expressing tumors or day 9 for C1GALT1 shRNA-expressing tumors), mice were treated daily with vehicle or ITZ (100 mg/kg by oral gavage) and tumor dimensions were monitored every 3 days. Data are presented as mean ± SD (*n* = 6 mice per group). *P* values were calculated following 15 days of treatment using mixed-effect analysis with post-hoc Tukey's multiple comparisons test. Source data are provided as a Source Data file.

Supplementary Table 1. List of the nine highest ranking validated factors identified from the screen, as well as representative other candidates of interest. P-values were calculated using a two-tailed Fisher's Exact test with Benjamini-Hochberg adjustment to control for the false discovery rate in the context of multiple comparisons. The P-value given is for the top-scoring sgRNA of the set. Source data are provided in Supplementary Data 1.

**Factors that promote EWSR1::FLI1 expression**				
**Biological process**	**Gene symbol**	**Gene name**	**Number of sgRNAs identified**	**P-value**
**Transcriptional regulation**	*H3C10*	H3 clustered histone 10	4	2.63E-80
	ZBTB21	Zinc finger and BTB domain containing 21	4	3.96E-63
	HIRA	Histone cell cycle regulator	4	1.50E-54
	ATF6B	Activating transcription factor 6 beta	4	1.08E-41
**Cell signaling**	*LAT*	Linker for activation of T cells	4	3.55E-54
	DOCK11	Dedicator of cytokinesis 11	4	1.70E-47
**Protein metabolism**	*C1GALT1*	Core 1 synthase, glycoprotein-N-acetylgalactosamine 3-beta-galactosyltransferase 1	4	7.90E-67
	FUT3	Fucosyltransferase 3	4	9.61E-58
	HMGCL	3-hydroxy-3 methylglutaryl-CoA lyase	4	6.64E-37
**Representative other candidates of interest**				
**EWSR1::FLI1 expression/stability**	*HNRNPH1*	Heterogeneous nuclear ribonuclear protein H1	2	1.74E-82
	SF3B1	Splicing factor 3b subunit 1	2	1.12E-20
	USP14	Ubiquitin specific peptidase 14	4	1.40E-07
	USP19	Ubiquitin specific peptidase 19	2	4.89E-04
**Hh signaling**	*GLI1*	GLI1 family zinc finger 1	3	1.61E-42
	SMO	Smoothened frizzled class receptor	2	2.55E-10
**C1GALT1 function**	*C1GALT1C1*	C1GALT1 specific chaperone1	3	6.22E-44
