# Supplementary material for: The O-glycosyltransferase C1GALT1 promotes EWSR1::FLI1 expression and is a therapeutic target for Ewing sarcoma
Source: Nat Commun. 2025 Feb 2;16:1267. doi: 10.1038/s41467-025-56632-0 (PMC11788431; doi:10.1038/s41467-025-56632-0)
Supplement: Supplementary file 2 — Description of Additional Supplementary Files [file 41467_2025_56632_MOESM2_ESM.pdf]

Description of Additional Supplementary Files

Supplementary Data 1. Complete results from the CRISPR/Cas9 screen. P-values were calculated using a two-tailed Fisher's Exact test with Benjamini-Hochberg adjustment to control for the false discovery rate in the context of multiple comparisons.

Supplementary Data 2. List of ~70 genes for which four sgRNAs were significantly enriched in the tdTomato^{low} EGFP^{high} population of the primary screen. P-values were calculated using a two-tailed Fisher's Exact test with Benjamini-Hochberg adjustment to control for the false discovery rate in the context of multiple comparisons. The P-value given is for the top-scoring sgRNA of the set.

Supplementary Data 3. List of primer sequences used for cloning, RT-PCR, qRT-PCR and ChIP, and shRNAs used in this study.
