# Supplementary material for: The O-glycosyltransferase C1GALT1 promotes EWSR1::FLI1 expression and is a therapeutic target for Ewing sarcoma
Source: Nat Commun. 2025 Feb 2;16:1267. doi: 10.1038/s41467-025-56632-0 (PMC11788431; doi:10.1038/s41467-025-56632-0)
Supplement: Supplementary file 6 — Reporting Summary [file 41467_2025_56632_MOESM6_ESM.pdf]

nature portfolio

Corresponding author(s): Shahid Banday; Sunil K. Malonia

Last updated by author(s): 01/22/2025

Reporting Summary

Nature Portfolio wishes to improve the reproducibility of the work that we publish. This form provides structure for consistency and transparency in reporting. For further information on Nature Portfolio policies, see our Editorial Policies and the Editorial Policy Checklist.

Statistics

For all statistical analyses, confirm that the following items are present in the figure legend, table legend, main text, or Methods section.

n/a	Confirmed	
☐	☒	The exact sample size (*n*) for each experimental group/condition, given as a discrete number and unit of measurement
☐	☒	A statement on whether measurements were taken from distinct samples or whether the same sample was measured repeatedly
☐	☒	The statistical test(s) used AND whether they are one- or two-sided<br>*Only common tests should be described solely by name; describe more complex techniques in the Methods section.*
☐	☒	A description of all covariates tested
☐	☒	A description of any assumptions or corrections, such as tests of normality and adjustment for multiple comparisons
☐	☒	A full description of the statistical parameters including central tendency (e.g. means) or other basic estimates (e.g. regression coefficient) AND variation (e.g. standard deviation) or associated estimates of uncertainty (e.g. confidence intervals)
☐	☒	For null hypothesis testing, the test statistic (e.g. *F*, *t*, *r*) with confidence intervals, effect sizes, degrees of freedom and *P* value noted<br>*Give P values as exact values whenever suitable.*
☒	☐	For Bayesian analysis, information on the choice of priors and Markov chain Monte Carlo settings
☒	☐	For hierarchical and complex designs, identification of the appropriate level for tests and full reporting of outcomes
☒	☐	Estimates of effect sizes (e.g. Cohen's *d*, Pearson's *r*), indicating how they were calculated

Our web collection on statistics for biologists contains articles on many of the points above.

Software and code

Policy information about availability of computer code

Data collection	NGS sequencing data were collected using an Illumina MiSeq System. qRT-PCR data was collected using a Quant Studio 3 real-time PCR system (Applied Biosystems by Thermo Scientific). Immunoblot data were collected using a Kodak X-OMAT 2000A film processor and ChemiDoc Imaging System (Bio-Rad). Cell viability and luciferase data were collected from a GlowMax plate reader (Promega). Confocal data were collected using a ZEISS LSM 900 confocal microscope equipped with ZEISS Zen Software. IHC data were collected using an Olympus BX40 microscope. Soft agar assay data were collected using a Zeiss Axio Observer equipped with ZEISS ZEN imaging software (v2.3). Cell counting data were collected using a Countess 3 FL Automated Cell Counter (Invitrogen) with Countess 3 FL software (v1.1.505.429).
Data analysis	Flow cytometry data were analyzed using FlowJo (v10). For the CRISPR screen, raw read quality was assessed using FastQC (v0.11.5), sequences were mapped using Bowtie (v1.2.2), and statistical analysis was performed using the CRISPRscreen R package available at https://doi.org/10.5281/zenodo.14226511. Densitometry of immunoblots was performed using ImageJ (v1.47). For GLI reporter assays, raw data (fluorescence readings) were analyzed in Microsoft Excel (v16.91). Microscope images were processed using ZEISS ZEN imaging software (v3.0). For Ewing sarcoma data analysis, datasets GSE17679 and GSE63157 were analyzed using a custom script available at https://doi.org/10.5281/zenodo.14226912. In silico GLI motif analysis was done using the Broad Institute's Integrative Genomics Viewer (IGV) genome browser (https://software.broadinstitute.org/software/igv/). Statistical analyses were performed with GraphPad Prism (v10.2.3).

For manuscripts utilizing custom algorithms or software that are central to the research but not yet described in published literature, software must be made available to editors and reviewers. We strongly encourage code deposition in a community repository (e.g. GitHub). See the Nature Portfolio guidelines for submitting code & software for further information.

Data

Policy information about availability of data

All manuscripts must include a data availability statement. This statement should provide the following information, where applicable:

- Accession codes, unique identifiers, or web links for publicly available datasets
- A description of any restrictions on data availability
- For clinical datasets or third party data, please ensure that the statement adheres to our policy

The raw sequencing data from the CRISPR/Cas9 screen have been deposited at the NCBI Gene Expression Omnibus under accession number GSE287721 [https: https://www.ncbi.nlm.nih.gov/geo/query/acc.cgi?acc=GSE287721]. This paper analyzed previously published RNA-seq data deposited at the NCBI Gene Expression Omnibus under accession numbers GSE17679 [https: https://www.ncbi.nlm.nih.gov/geo/query/acc.cgi?acc=GSE17679] and GSE63157 [https://www.ncbi.nlm.nih.gov/geo/query/acc.cgi?acc=GSE63157]. Datasets GSE17679 and GSE63157_core_transcriptom were downloaded from the R2 platform [R2:GenomicsAnalysis and Visualization Platform (http://r2.amc.nl)]. The remaining data supporting the findings of this study are available within the Article, Supplementary Information or Source Data. Source data are provided with this paper.

Research involving human participants, their data, or biological material

Policy information about studies with human participants or human data. See also policy information about sex, gender (identity/presentation), and sexual orientation and race, ethnicity and racism.

Reporting on sex and gender	N/A
Reporting on race, ethnicity, or other socially relevant groupings	N/A
Population characteristics	N/A
Recruitment	N/A
Ethics oversight	N/A

Note that full information on the approval of the study protocol must also be provided in the manuscript.

Field-specific reporting

Please select the one below that is the best fit for your research. If you are not sure, read the appropriate sections before making your selection.

☒ Life sciences ☐ Behavioural & social sciences ☐ Ecological, evolutionary & environmental sciences

For a reference copy of the document with all sections, see nature.com/documents/nr-reporting-summary-flat.pdf

Life sciences study design

All studies must disclose on these points even when the disclosure is negative.

Sample size	No statistical methods were used to predetermine the sample size. For the majority of in vitro quantitative experiments (except Fig. 5d), sample sizes were n=3-5 independent experiments, and each experiment comprised three technical replicates, which is standard practice and considered the minimum necessary to obtain statistically reliable results and account for biological variability. For in vivo experiments, n=5-6 mice per group were taken as per standard practice in the field. Sample sizes were consistent within each experiment.
Data exclusions	No data were excluded from the analyses.
Replication	Data for all qRT-PCR, ChIP, luciferase reporter, cell viability and colony formation assays were collected from 3-5 independent biological replicates as stated in the figure legends (and each independent experiment included at least three technical replicates of the sample). The majority of the immunoblot experiments were conducted three independent times, with the exception of Supp Figs. 1f, 4b, 4c, 5b, 6c, 6e, and 9a-c, which were conducted twice; and Supp Figs. 3d, 3f, and 3g, which were conducted once. The PNA lectin pull-down assays of Fig. 5a and Supp Fig. 11a were performed three independent times and the JAC lectin pull-down assay of Supp Fig. 5a was performed twice. The co-immunoprecipitation experiments of Fig. 5b were performed three independent times. The cycloheximide chase assay of Fig. 5d was performed two independent times. The ubiquitination assay of Fig. 5h was performed three independent times. The RT-PCR experiment of Supp Fig. 1b was performed once. The flow cytometry analysis of Supp Fig. 1c was performed two independent times, and Supp Fig. 1d was performed three independent times. The immunofluorescence assay of Supp Fig. 5c was performed twice. For all IHC analyses, at least three sections of each tumor sample were analyzed. All attempts at replication were successful.
Randomization	For in vivo studies, mice were randomly allocated to each group. For in vitro experiments, randomization was not used for group allocation because these experiments required controlled conditions and specific variables to define the experimental setup.
Blinding	Investigators were not blinded for any of the experiments, including to the animal assignments for tumor formation studies and molecular analysis, as treatment conditions were evident from the data. Animal groups were identified by tagging and labeling the cages with the cells injected.

Reporting for specific materials, systems and methods

We require information from authors about some types of materials, experimental systems and methods used in many studies. Here, indicate whether each material, system or method listed is relevant to your study. If you are not sure if a list item applies to your research, read the appropriate section before selecting a response.

Materials & experimental systems

n/a	Involved in the study
☐	☒ Antibodies
☐	☒ Eukaryotic cell lines
☒	☐ Palaeontology and archaeology
☐	☒ Animals and other organisms
☒	☐ Clinical data
☒	☐ Dual use research of concern
☒	☐ Plants

Methods

n/a	Involved in the study
☒	☐ ChIP-seq
☐	☒ Flow cytometry
☒	☐ MRI-based neuroimaging

Antibodies

Antibodies used

Primary antibodies used for immunoblotting include: anti-C1GALT1 (clone F-31) (1:1000 dilution; Santa Cruz Biotechnology, Cat# sc-100745), anti-EWSR1 (1:1000 dilution; Sigma Aldrich, Cat# HPA051771), anti-FLI1 (clone EPR4646) (1:2000 dilution; Abcam, Cat# ab133485), anti-GLI1 (1:1000 dilution; Cell Signaling Technology, Cat# 2553), anti-GLI2 (clone OTI1F9) (1:500 dilution; Abcam, Cat# ab187386), anti-SMO (1:1000 dilution; Abcam, Cat# ab236465), anti-PARP1(clone F-2) (1:500 dilution; Santa Cruz Biotechnology, Cat# sc-8007), anti-CYP51A1 (clone N6-P2H5*G8) (1:1000 dilution; EMD Millipore, Cat# MABS1259), anti-CYP3A4 (clone HL3) (1:500 dilution; Santa Cruz Biotechnology, Cat# sc-53850), anti-pAKT (Ser473) (1:1000 dilution; Cell Signaling Technology, Cat# 9271T), anti-AKT (1:1000 dilution; Cell Signaling Technology, Cat# 9272S), anti-pS6K(Thr389) (clone 108D2) (1:1000 dilution; Cell Signaling Technology, Cat#9234T), anti-S6K (1:1000; Cell Signaling Technology, Cat# 9202S), anti-beta-catenin (clone D10A8) (1:1000 dilution; Cell Signaling Technology, Cat# 8480T), anti-beta-actin (clone AC-74) (1:2000 dilution; Sigma, Cat# A2228), anti-GAPDH (clone 6C5) (1:2000 dilution; Abcam, Cat# ab8245), and anti-FLAG (clone M2) (1:1000 dilution; Sigma, Cat# F1804). Secondary antibodies for western blotting include rabbit IgG, HRP-linked whole Ab (1:5000 dilution; Cytiva, Cat# NA934V) or mouse IgG, HRP-linked whole Ab (1:4000 dilution; Cytiva, Cat# NA931V), HRP Streptavidin (1:6000; Biolegend, Cat # 405210).

Antibodies for ChIP assays include: anti-GLI1 antibody (5 ug; R&D Systems, Cat#: AF3324), anti-GLI2 antibody (5 ug; R&D Systems, Cat#: AF3526).

Antibodies used for IF include: anti-FLAG (clone M2) (1:500 dilution; Sigma, Cat# F1804), and F(ab')2-Goat anti-Mouse IgG (H+L) Cross-Adsorbed Secondary Antibody, Alexa Fluor 647 (1:1000 dilution; ThermoFisher Scientific, Cat# A21237).

Antibodies used for ubiquitination assays include: anti-FLAG (clone M2) (1:1500 dilution; Sigma, Cat# F1804) and anti-HA (clone 6E2) (1:1500 dilution; Cell Signaling Technology, Cat# 2367) antibody.

Antibodies used for IHC include: anti-FLI1 (clone EPR4646) (1:50 dilution; Abcam, Cat# ab133485), anti-EWSR1 (1:50 dilution; Sigma Aldrich, Cat# HPA051771), anti-Ki67 (1:250 dilution; Sino Biological, Cat# 100130-MM22; reacts with both human and mouse proteins), anti-C1GALT1 (clone F-31) (1:100 dilution, Santa Cruz Biotechnology, Cat# sc-100745), anti-SMO (1:50, Abcam, Cat# ab236465), anti-GLI1 (clone OTI2E1) (1:50 dilution, Thermo Fisher Scientific, Cat# MA5-26639), anti-FLAG (clone 9A3) (1:250 dilution; Cell Signaling Technology, Cat#8146T).

Validation

All commercially available antibodies were validated as stated on manufacturer's or supplier's data sheets:
1) anti-C1GALT1 (Santa Cruz Biotechnology, Cat# sc-100745) https://www.scbt.com/p/c1galt1-antibody-f-31
2) anti-EWSR1 (Sigma Aldrich, Cat# HPA051771) https://www.sigmaaldrich.com/US/en/product/sigma/hpa062953
3) anti-FLI1 (Abcam, Cat# ab133485) https://www.abcam.com/en-us/products/primary-antibodies/fli1-antibody-epr4646-ab133485
4) anti-GLI1 (Cell Signaling Technology, Cat# 2553) https://www.cellsignal.com/products/primary-antibodies/gli1-antibody/2553
5) anti-GLI2 (Abcam, Cat# ab187386) https://www.abcam.com/en-us/products/primary-antibodies/gli2-antibody-oti1f9-ab187386
6) anti-SMO (Abcam, Cat# ab236465) https://www.abcam.com/en-us/products/primary-antibodies/smoothened-antibody-ab236465#
7) anti-PARP1 (Santa Cruz Biotechnology, Cat# SC-8007) https://www.scbt.com/p/parp-1-antibody-f-2
8) anti-CYP51A1 (EMD Millipore, Cat# MABS1259) https://www.sigmaaldrich.com/US/en/product/mm/mabs1259
9) anti-CYP3A4 (Santa Cruz Biotechnology, Cat# sc-53850) https://www.scbt.com/p/cyp3a4-antibody-hl3
10) anti-pAKT (Ser473) (Cell Signaling, Cat# 9271T) https://www.cellsignal.com/products/primary-antibodies/phospho-akt-ser473-antibody/9271
11) anti-AKT (Cell Signaling, Cat# 9272S) https://www.cellsignal.com/products/primary-antibodies/akt-antibody/9272
12) anti-pS6K(Thr389) (Cell Signaling, Cat#9234T) https://www.cellsignal.com/products/primary-antibodies/phospho-p70-s6-kinase-thr389-108d2-rabbit-mab/9234 12)
13) anti-S6K (Cell Signaling, Cat# 9202S) https://www.cellsignal.com/products/primary-antibodies/p70-s6-kinase-antibody/9202
14) anti-beta-catenin (Cell Signaling, Cat# 8480T) https://www.cellsignal.com/products/primary-antibodies/b-catenin-d10a8-xp-rabbit-mab/8480
15) anti-beta-actin (Sigma, Cat# A2228) https://www.sigmaaldrich.com/US/en/product/sigma/a2228
16) anti-GAPDH (Abcam, Cat# ab8245) https://www.abcam.com/en-us/products/primary-antibodies/gapdh-antibody-6c5-loading-control-ab8245
17) anti-FLAG M2 (Sigma, Cat# F1804) https://www.sigmaaldrich.com/US/en/product/sigma/f1804

18) anti-GLI1 antibody (R&D Systems, Cat#: AF3324) https://www.rndsystems.com/products/human-gli-2-antibody_af3526
19) anti-GLI2 antibody (R&D Systems, Cat#: AF3526) https://www.rndsystems.com/products/human-gli-2-antibody_af3526
20) anti-Ki67 (Sino Biological, Cat# 100130-MM22)
https://www.sinobiological.com/antibodies/human-ki67-mki67-100130-mm22
21) anti-GLI1 (ThermoFisher Scientific, Cat# MA5-26639) https://www.thermofisher.com/antibody/product/GLI1-Antibody-clone-OTI2E1-Monoclonal/MA5-26639
22) anti-FLAG (Cell Signaling, Cat#8146T) https://www.cellsignal.com/products/primary-antibodies/dykddddk-tag-9a3-mouse-mab-binds-to-same-epitope-as-sigma-aldrich-anti-flag-m2-antibody/8146
23) anti-HA (Cell Signaling Cat# 2367) https://www.cellsignal.com/products/primary-antibodies/ha-tag-6e2-mouse-mab/2367
24) F(ab')2-Goat anti-Mouse IgG (H+L) Cross-Adsorbed Secondary Antibody, Alexa Fluor 647 (ThermoFisher Scientific, Cat# A21237) https://www.thermofisher.com/antibody/product/Goat-anti-Mouse-IgG-H-L-Cross-Adsorbed-Secondary-Antibody-Polyclonal/A-21237
25) Rabbit IgG, HRP-linked whole Ab (Cytiva, Cat# NA934V) https://www.cytivalifesciences.com/en/us/shop/protein-analysis/blotting-and-detection/blotting-standards-and-reagents/amersham-ecl-hrp-conjugated-antibodies-p-0626026)
26) Mouse IgG, HRP-linked whole Ab (Cytiva, Cat# NA931V) https://www.cytivalifesciences.com/en/us/shop/protein-analysis/blotting-and-detection/blotting-standards-and-reagents/amersham-ecl-hrp-conjugated-antibodies-p-06260
27) HRP Streptavidin (Biolegend, Cat # 405210) https://www.biolegend.com/en-us/products/hrp-streptavidin-1474

Eukaryotic cell lines

Policy information about cell lines and Sex and Gender in Research

Cell line source(s)	A673 (CRL-1589; female), SK-N-MC (HTB-10; female), IMR-90 (CCL-186; female), NIH 3T3 (CRL-1658), and HepG2 (HB-8065; male) cell lines were obtained from the American Type Culture Collection (ATCC), and TC-32 (female), TC-71 (male) and TC-106 (male) were obtained from The Childhood Cancer Repository at Texas Children's Hospital.
Authentication	All parental cell lines obtained from the ATCC were verified based on the information provided by the supplier. Cell lines were routinely monitored for phenotypes such as morphology and growth kinetics. The CRISPR knock-in reporter cell line was validated by PCR, Sanger sequencing and flow cytometry. CRISPR edited cell lines were validated by Sanger sequencing. Stable shRNA-mediated knockdown cell lines were validated by qRT-PCR and immunoblot analyses.
Mycoplasma contamination	Cell lines were mycoplasma negative and were routinely tested for mycoplasma contamination.
Commonly misidentified lines (See ICLAC register)	SK-N-MC was originally identified as a neuroblastoma cell line but has been reclassified as a Ewing sarcoma cell line (Staege et al., 2004; PMID 15548687).

Animals and other research organisms

Policy information about studies involving animals; ARRIVE guidelines recommended for reporting animal research, and Sex and Gender in Research

Laboratory animals	6–7-week-old male or female NOD-scid IL2Rgammanull (NSG) mice (Jackson Laboratory, Strain# 005557)
Wild animals	The study did not involve wild animals.
Reporting on sex	Sex was not considered in the study design; ES affects both males and females. and therefore both male and female mice were used.
Field-collected samples	The study did not involve samples collected from the field.
Ethics oversight	All animal experiments were performed in accordance with the Guide for the Care and Use of Laboratory Animals from NIH, and a protocol (PROTO202000105) approved by the UMass Chan Medical School Institutional Animal Care and Use Committee (IACUC).

Note that full information on the approval of the study protocol must also be provided in the manuscript.

Plants

Seed stocks	N/A
Novel plant genotypes	N/A
Authentication	N/A

Flow Cytometry

Plots

Confirm that:

☒ The axis labels state the marker and fluorochrome used (e.g. CD4-FITC).

☒ The axis scales are clearly visible. Include numbers along axes only for bottom left plot of group (a 'group' is an analysis of identical markers).

☒ All plots are contour plots with outliers or pseudocolor plots.

☒ A numerical value for number of cells or percentage (with statistics) is provided.

Methodology

Sample preparation	A673/EWSR1::FLI1tdTomato/EGFP/Cas9 reporter cells transduced with the Human CRISPR Knockout Pooled Library (Brunello) (Addgene, Cat#73178) were selected with 2 µg/ml puromycin for 12 days, followed by 3 days recovery in the absence of puromycin. 1E8 cells were subjected to FACS sorting.
Instrument	Cell sorting was performed using cell sorter BD FACSAria II. Flow analysis was performed on BD LSR II Flow Cytometer
Software	Post acquisition data was analyzed using FlowJo v10 (BD Biosciences)
Cell population abundance	N/A
Gating strategy	Total live cells were gated using FSC-A/SSC-A to exclude debris and dead cells. Single cells were identified by gating on FSC-H versus FSC-A to eliminate doublets and aggregates. From the singlet population, GFP-positive and tdTomato-positive cells were gated based on fluorescence intensity. Subsequently, a subset of cells with low tdTomato expression and high GFP expression was selected for sorting.

☒ Tick this box to confirm that a figure exemplifying the gating strategy is provided in the Supplementary Information.
